# Functional connectivity analysis on electroencephalography signals reveals potential biomarkers for treatment response in major depression

**DOI:** 10.1186/s12888-023-04958-8

**Published:** 2023-08-01

**Authors:** Shiau-Shian Huang, Yu-Hsiang Yu, His-Han Chen, Chia-Chun Hung, Yao-Ting Wang, Chieh Hsin Chang, Syu-Jyun Peng, Po-Hsiu Kuo

**Affiliations:** 1 Department of Medical Education, Taipei Veterans General Hospital, Taipei, Taiwan; 2 Institute of Epidemiology and Preventive Medicine, College of Public Health, National Taiwan University, Taipei, Taiwan; 3 College of Medicine, National Yang Ming Chiao Tung University, Taipei, Taiwan; 4 Bali Psychiatric Center, Ministry of Health and Welfare, New Taipei, Taiwan; 5 Division of Neurology, Taipei Veterans General Hospital, Taipei, Taiwan; 6 Department of Psychiatry, Yang Ji Mental Hospital, Keelung, Taiwan; 7 Program in Artificial Intelligence in Medicine, College of Medicine, Taipei Medical University, Taipei, Taiwan; 8 Department of Psychiatry, National Taiwan University Hospital, Taipei, Taiwan; 9 Psychiatric Research Center, Wan Fang Hospital, Taipei Medical University, Taipei, Taiwan

**Keywords:** Major depression, Electroencephalography, Functional connectivity, Antidepressants, Treatment response

## Abstract

**Background:**

The treatment efficacy varies across individual patients with major depressive disorder (MDD). It lacks robust electroencephalography (EEG) markers for an antidepressant-responsive phenotype.

**Method:**

This is an observational study enrolling 28 patients with MDD and 33 healthy controls (mean age of 40.7 years, and 71.4% were women). Patients underwent EEG exams at baseline (week0) and week1, while controls’ EEG recordings were acquired only at week0. A resting eye-closing EEG segment was analyzed for functional connectivity (FC). Four parameters were used in FC analysis: (1) node strength (NS), (2) global efficiency (GE), (3) clustering coefficient (CC), and (4) betweenness centrality (BC).

**Results:**

We found that controls had higher values in delta wave in the indices of NS, GE, BC, and CC than MDD patients at baseline. After treatment with antidepressants, patients’ FC indices improved significantly, including GE, mean CC, and mean NS in the delta wave. The FC in the alpha and beta bands of the responders were higher than those of the non-responders.

**Conclusions:**

The FC of the MDD patients at baseline without treatment was worse than that of controls. After treatment, the FC improved and was close to the values of controls. Responders showed better FC in the high-frequency bands than non-responders, and this feature exists in both pre-treatment and post-treatment EEG.

**Supplementary Information:**

The online version contains supplementary material available at 10.1186/s12888-023-04958-8.

## Background

Major depressive disorder (MDD) is a common mental illness with a high lifetime prevalence [1]. The symptoms of MDD significantly impair patients’ daily functions [1]. Selective serotonin reuptake inhibitors (SSRIs) are recommended as the first-line treatment for depression [2]. However, the treatment efficacy with SSRIs varies widely across patients, and the clinical response rate ranges between one-third to two-thirds [3, 4] due to the heterogeneity in clinical presentations and genetic predisposition [5]. Previous studies using genetic and serum biomarkers did not capture large variation in treatment response, neither reflect brain signals directly [6, 7]. In comparison, non-invasive and safe electroencephalogram (EEG) is easier to capture real-time and direct brain signals. The EEG biomarkers might help dissect the biological underpinnings of clinical manifestations and tailor treatment prescription [8].

EEG can reveal oscillations emanating from the brain in characteristic frequency bands, such as the power values of the theta (4–8 Hz), alpha (8–13 Hz), and beta (13–30 Hz). EEG signals were found to distinguish MDD patients and healthy controls [9]. Moreover, previous EEG studies using spectrum analysis (SA) reported predictive value for treatment response [9, 10]. However, the potential signals from spectrum EEG analysis could not provide a robust prediction at the patient level [11, 12]. On the other hand, MDD is increasingly recognized as a disorder with dysregulated neural networks rather than a local brain disorder [13]. Power SA is considered to reveal the strength of the local signals but does not sufficiently reflect distributed networks related to mood.

One recent systematic review has suggested using functional connectivity (FC) analysis to help reveal the pattern changes of different activities in a depressed patient [14]. FC analysis refers to the observed connection between interconnected brain areas [15, 16]. Studies used complex network analysis has its origins in the mathematical study of networks, known as graph theory [17]. A graph is an abstract representation of a network and it consists of a set of nodes and connections (edges) [17]. Brain connectivity datasets comprise networks of brain regions that are connected by anatomical tracts or functional associations [15, 17, 18]. Dissecting functional network topologies among patients reveal the presumed connectivity abnormalities in neurological and psychiatric disorders compared with healthy controls [19, 20].

A resting EEG study found that FC between the prefrontal cortex and posterior cingulate cortex is elevated in remitted MDD, suggesting EEG FC as a neural marker of depression [21]. One clinical study found a negative relationship between FC and depressive severity [22]. A previous magnetic resonance imaging (MRI) study showed FC changes following antidepressant medication, for which increased connectivity between frontal and limbic brain regions was reported [23]. Another EEG study on Alzheimer’s disease showed the advantages of the FC analysis over the traditional SA method [24]. Therefore, EEG FC may have the potential to evaluate its predictive power for the treatment response of antidepressants in MDD. Delineating the FC for depression would advance a neurobiological understanding of treatment response and assist in identifying patients who benefit from medication. So far, it lacks studies investigating the predictive performance of EEG, which can compare the results between SA and FC for MDD diagnosis and treatment response. There were several aims in the present study. First, we explored the differences in FC between MDD patients and healthy controls. We also evaluated the differences in FC among MDD patients before and after treatment (the combined effects of antidepressants and therapeutic effects). Second, previous EEG studies have suggested that early EEG changes may correlate with clinical responses [25]. We hypothesized that the changes in graph-theoretical brain FC in the first week of treatment could serve as markers for evaluating the effectiveness of antidepressants treatment at the week4. Third, we investigated the differences in FC between responders and non-responders (therapeutic effects). Fourth, we explored the correlation between depressive severity at baseline and EEG FC at baseline. Finally, the discriminative ability of MDD diagnosis and treatment response was investigated using band power values and FC analyses.

## Materials and methods

### Study design and participants

This is a clinical observational EEG study for depression. The study flowchart is presented in Supplementary Fig. 1. From January 2019 to December 2021, patients with MDD diagnosis, according to the Diagnostic and Statistical Manual of Mental Disorders, 5th edition, were enrolled in psychiatric clinics in Taiwan. All patients were at least 16 years old and interviewed by board-certified psychiatrists and trained research nurses. Inclusion criteria included patients with a depressive episode at the baseline of at least 14 points rated by the 17-item Hamilton Rating Scale for Depression (HAM-D) [26]. All participants were free of antidepressant medications for at least 7–10 days before enrollment. Patients then received antidepressant treatment according to the psychiatrist’s clinical judgment. Healthy controls were free of lifetime psychiatric illness or substantial medical conditions. All participants were free of active infections or systematic diseases as confirmed by medical history and a complete chart review system. The exclusion criteria of participants are shown in the Supplementary Materials.

### Study assessments and outcome

Participants were assessed using HAM-D at week 0, week1, week2, week4, and week8. A higher score indicates more severe depressive symptoms. A trained lay interviewer rated HAM-D to obtain information on depressive severity. The inter-rater reliability reached 0.84 [26]. Participants were also assessed using the Young mania rating scale at each time point to exclude the possibility of bipolar disorder. Clinical response (≥ 50% improvement in HAM-D scores) was examined for each subject at week1, week4, and week8. Short-term response (at week4) was used to define treatment responders or non-responders.

### EEG Recording

Patients underwent an EEG exam with 19 electrodes at both w0 and w1, while healthy controls’ EEG recordings were acquired only at w0. EEG exams were sampled at 256–500 Hz. EEG activity was recorded using 19 electrodes (Nicolette V32) referenced to the Cz electrode and positioned according to the 10–20 international electrode placement system. EEG was recorded during 17-minute periods (including eyes-closed, eyes-opening, deep breathing, intermittent photic stimulation, looking sad picture, and looking happy picture) (Supplementary Fig. 2). Participants were instructed to remain still, awake, minimize blinks or eye movements, and fixate on a centrally presented cross during the eyes-open condition.

### EEG Preprocessing

EEG data were preprocessed by MATLAB R2019. A segment of a one-minute eye-closing was selected. Data were re-referenced to the mean of all scalp channels to reduce the common effect of each channels. An independent component analysis (ICA) decomposition was performed [27] to remove EEG eye movements and other mechanical artifacts. The relative power was calculated using continuous wavelet transform (CWT) across all electrodes (Fp1, Fp2, F3, F4, C3, C4, P3, P4, O1, O2, F7, F8, T3, T4, T5, T6, Fz, Cz, and Pz) for delta (0.5–4 Hz), theta (4–8 Hz), alpha (8–13 Hz), and beta (13–30 Hz) frequency bands [28].

### FC and phase locking value

FC was estimated with phase locking value (PLV) to explore the FC across broad brain regions. PLV measure is a well-known method for phase synchronization quantitation [29]. The PLV ranges between zero and one. A “zero“ value indicates no coupling occurs, and a value of “one” tells perfect phase locking.

### Graph theoretical analysis (GTA)

Graph theory is a method that can be applied to brain networks with the calculation of PLV. A graph consists of nodes and connections [17]. Weighted connectivity matrices were obtained by applying a series of thresholds to the 19 × 19 weighted adjacency matrices of PLV for each subject and frequency band. The threshold points were set to the 90th, 80th, …, and 10th percentiles of the matrices, resulting in 9 matrices with densities of 10%, 30%, …, and 90%. Threshold points of 10% and 90% were removed to diminish the impact of extreme settings. The weighted matrices were analyzed using indexes based on the graph theory [30]. To illustrate a network measure, we consider a basic and important measure known as degree [31]. The degree refers to the number of connections that a node has in a network. Node strength (NS) is a more complex measure that takes into account the number of connections a node has, and the strength or weight of those connections [31]. Four parameters were estimated using Brain Connectivity Toolbox (algorithm formulas were shown in Supplementary Table 1) [31]: (1) NS, (2) measures of integration [global efficiency (GE)], (3) measures of segregation [clustering coefficient (CC)], and (4) measures of centrality based on node degree or on the length and number of shortest paths between nodes [betweenness centrality (BC)].

### Statistical analysis

A *t*-test and chi-square test were used to determine the demographic differences between patients and controls. Wilcoxon rank-sum and sign-rank tests were used to determine the groups’ relative power differences and FC parameters. Results were assessed with correction for multiple comparisons using the false discovery rate [32]. The correlation between change in FC and change in HAM-D was evaluated by using Spearman’s rank correlation adjusted with age.

## Results

### Demographic and clinical data among participants

There were 28 MDD patients and 33 controls. There were no significant differences in demographic features between patients and controls (Table 1). Patients drank alcohol more frequently, had lower education degrees, and had less regular exercise. Patients were treated with escitalopram (75.00%), sertraline (7.14%), bupropion (7.14%), agomelatine (7.14%) or paroxetine (3.57%). Around half of the participants (48%) have had previous suicide attempts. The total score of HAM-D was high at baseline (19.32 ± 3.52) and gradually decreased during follow-up (week8: 7.09 ± 7.56).

**Table 1:** Demographic and clinical characteristics of patients and control in the EEG study

	Control		Patients with MDD		p
Variables	n	%	n	%	
Total (N = 61)	33	54.10	28	45.90	
Female	28	84.85	20	71.43	0.202
Marriage or in a long-term relationship	9	27.27	10	37.04	0.419
The habit of smoking	6	18.18	5	20^#^	0.861
The habit of drinking alcohol*	1	3.03	5	20^#^	< 0.05
University degree or above*	19	57.58	8	29.63^#^	< 0.05
Job or employment	31	93.94	22	78.57	0.076
Religion	25	75.76	14	73.68^#^	0.868
Income					0.790
0	8	24.24	6	21.43	
1-30000	8	24.24	9	32.14	
> 30,000	17	51.52	13	46.43	
Satisfaction of sleep quality	10	30.3	8	29.63^#^	0.955
Baseline with using BZD	0	0	18	64.29	
Insight of disease	-	-	22	88^#^	
Family history of MDD	0	0	8	32^#^	
Ever attempt suicide	0	0	12	48.00^#^	
Right-hand dominance	33	100	28	100	1
Exercise habits*	26	78.79	10	38.46^#^	< 0.01
Variables	Mean	SD	Mean	SD	
Age	35.01	13.03	40.71	18.21	0.160
Number of prior depressive episodes	0	0	2.48	1.05	
Body Mass Index	22.04	3.02	22.61	3.50	0.495
Antidepressant					
Escitalopram	-	-	21	75.00	
Sertraline	-	-	2	7.14	
Bupropion	-	-	2	7.14	
Agomelatine	-	-	2	7.14	
Paroxetine	-	-	1	3.57	
HAM-D-17 total score					
Baseline	-	-	19.32	3.52	
1-Week	-	-	12.21	4.90	
2-Week	-	-	11	5.44	
4-Week	-	-	10.04	6.39	
6-Week	-	-	8.93	6.71	
8-Week	-	-	7.09	7.56	

Abbreviation: SD, standard deviation, HAM-D, Hamilton depression rating scale, BZD, benzodiazepine.

*: Statistically significant difference

Note:^#^ indicates the variables with missing data of 1–3 individuals, except for Religion which has missing data in 9 individuals.

The demographic and clinical characteristics of the non-responders and responders (46.43%) are shown in Table 2. There were no significant differences in most clinical features between the two groups. A more significant proportion of patients in the non-responsive group used benzodiazepine (BZD) at enrollment (p = 0.0079). There was no significant difference in HAM-D scores at baseline between the two groups. It was not until two weeks later that there was a significant difference in HAM-D scores between the responsive and non-responsive groups.

**Table 2:** Demographic and clinical characteristics of the non-responsive and responsive group

	Non-responsive group		Responsive group		p
Variables	n	%	n	%	
Total (N = 28)	15	53.57	13	46.43	
Female	10	66.67	10	76.92	0.549
Marriage or in a long-term relationship	6	42.86^#^	4	30.77	0.516
The habit of smoking	6	50^#^	6	46.15	0.109
The habit of drinking alcohol	9	75^#^	11	84.62	0.548
University degree or above	5	35.71^#^	3	23.08	0.472
Job or employment	10	66.67	12	92.31	0.099
Religion	8	66.67^#^	6	85.71^#^	0.363
Income					0.978
0	3	20	3	23.08	
1-30000	5	33.33	4	30.77	
> 30,000	7	46.67	6	46.15	
Satisfaction of sleep quality	4	28.57^#^	4	30.77	0.901
Baseline with using BZD*	13	86.67	5	38.46	< 0.01
Insight of disease	10	83.33^#^	12	92.31	0.490
Family history of MDD	3	25^#^	5	38.46	0.471
Ever attempt suicide	6	42.86^#^	6	46.15	0.360
Right-hand dominance	15	100	13	100	1
Exercise habits	4	30.77^#^	6	46.15	0.420
Variables	Mean	SD	Mean	SD	
Age	41.96	17.96	39.27	19.11	0.704
Number of prior depressive episodes	2.08	0.79	2.85	1.14	0.067
Body Mass Index	22.23	3.98	23.03	3.00	0.561
Antidepressant					0.295
Escitalopram	11	73.33	10	76.92	
Sertraline	1	6.67	1	7.69	
Bupropion	0	0	2	15.38	
Agomelatine	2	13.33	0	0	
Paroxetine	1	6.67	0	0	
Daily dose of antidepressants (DDD)	1.07	0.37	1.12	0.30	0.709
HAM-D-17 total score					
Baseline	18.93	3.20	19.77	3.94	0.541
1-Week	13.20	3.69	11.08	5.96	0.261
2-Week*	13.87	4.69	7.69	4.35	< 0.01
4-Week*	14.07	4.15	5.38	5.30	< 0.0001
6-Week*	12.93	5.88	4.62	4.61	< 0.001
8-Week*	11.30	6.20	3.58	6.93	< 0.05

Abbreviation: SD, standard deviation, HAM-D, Hamilton depression rating scale, BZD, benzodiazepine, DDD, defined daily dose.

*: Statistically significant difference.

Note:^#^ indicates the variables with missing data of 1–3 individuals, except for Religion which has missing data in 6 individuals.

### The difference in scalp networks between controls, patients at baseline

**Fig. 1:**
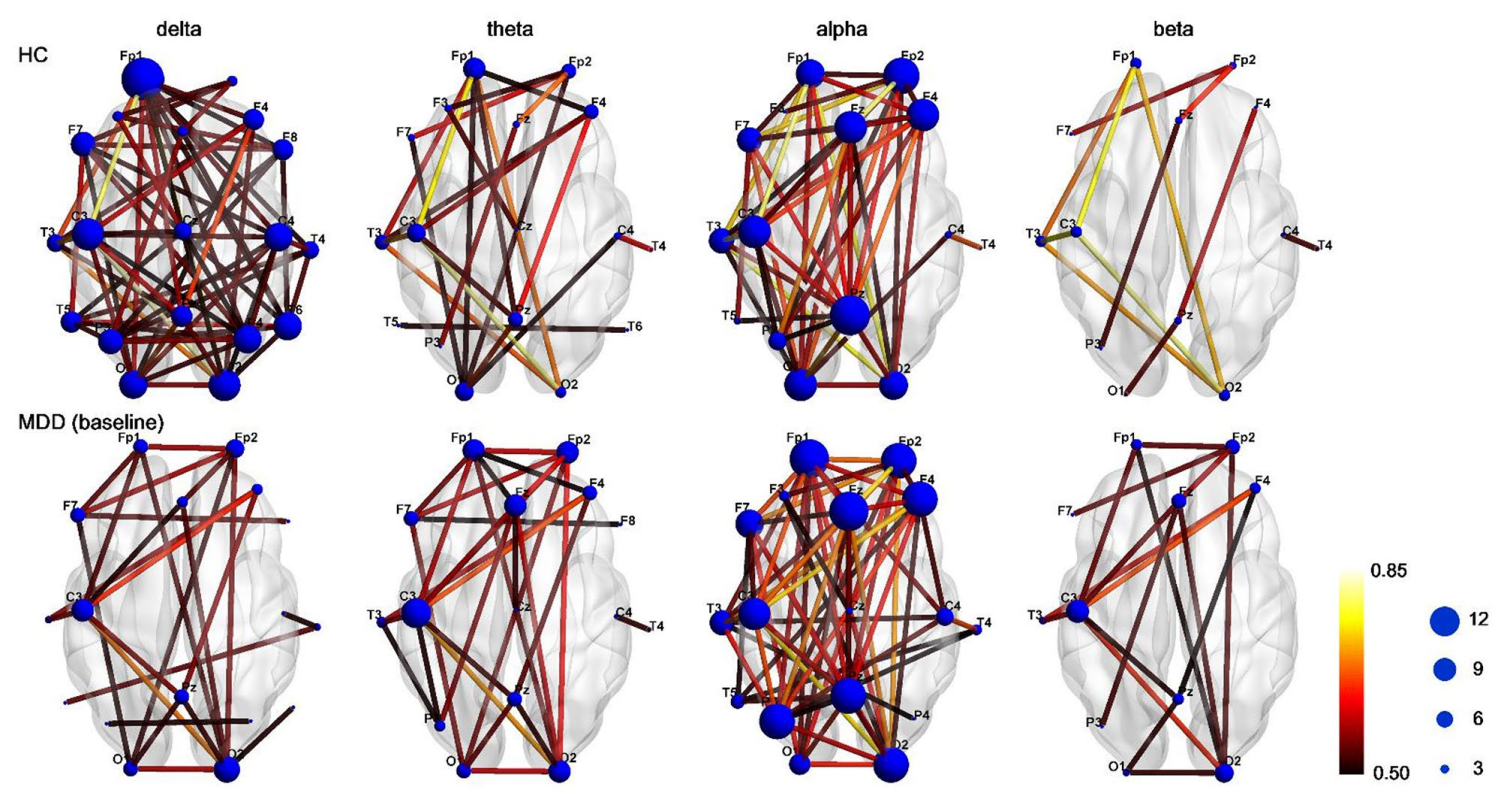
The difference in scalp networks between healthy controls (HC), MDD patients at baseline. Regional brain connectivity was depicted by weighted connectivity matrices in different frequency bands between groups. Line color represented average phase locking value (PLV) between each pair of the channels across all subjects in each group. The size of node represented numbers of connections in each node. The scalp networks in delta band in HC has more connections (PLV > 0.5) than those in MDD patients, and the node degree of each electrode is also greater in MDD patients

Regional brain connectivity was depicted by weighted connectivity matrices in different frequency bands between controls and patients at baseline (Fig. 1). The connectivity matrices for controls and MDD patients illustrated the weight of connection between electrodes. The scalp networks in delta band in controls has more connections (PLV > 0.5) than those in MDD patients, and the node degree of each electrode is also greater in MDD patients.

### The difference in FC between controls, patients before and after treatment

**Fig. 2:**
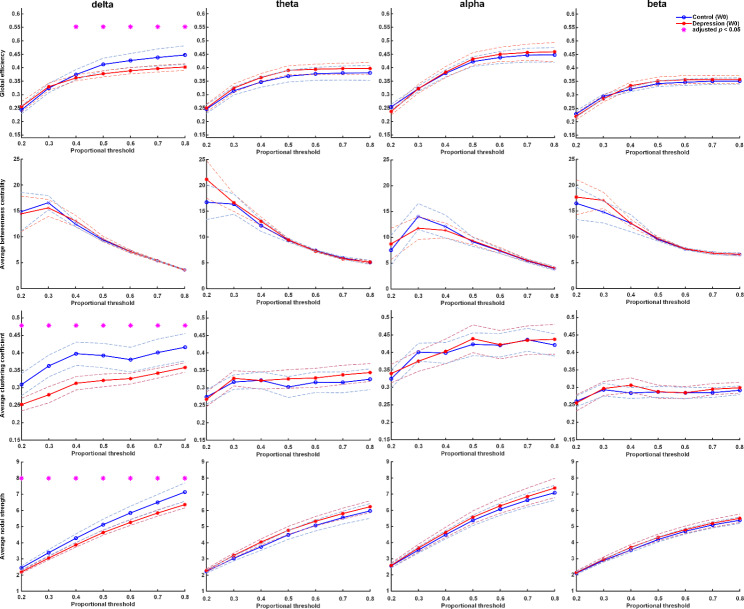
Graph theory-based analyses between patients at baseline and healthy controls. The figure shows functional connectivity with four parameters: global efficiency, mean clustering coefficient, mean node strength, and mean betweenness centrality in delta, theta, alpha, and beta frequency bands. Healthy controls had higher functional connectivity in the delta band than patients with major depression. Asterisks denote statistically significant differences (*: adjusted p under correction for multiple comparisons using the false discovery rate; dashed lines represent the 95% confidence interval)

**Fig. 3:**
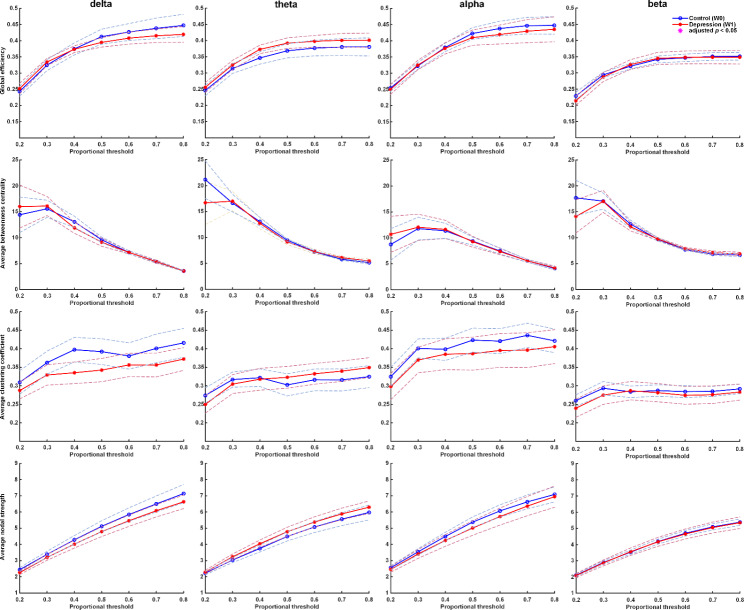
Graph theory-based analyses between patients at week-1 and healthy controls

Controls had higher values in delta wave in NS, mean GE, and mean CC compared with untreated patients at baseline (Fig. 2). After one-week treatment with antidepressants, patients’ FC improved significantly, including GE, mean CC, and mean NS in the delta wave (p-values < 0.05). Compared with controls, patients after one-week treatment had improved FC in delta wave, including GE, mean CC, and mean NS (Fig. 3). The FC of patients after one week of treatment was close to the FC of controls.

### The differences in FC between responders and non-responders

**Fig. 4:**
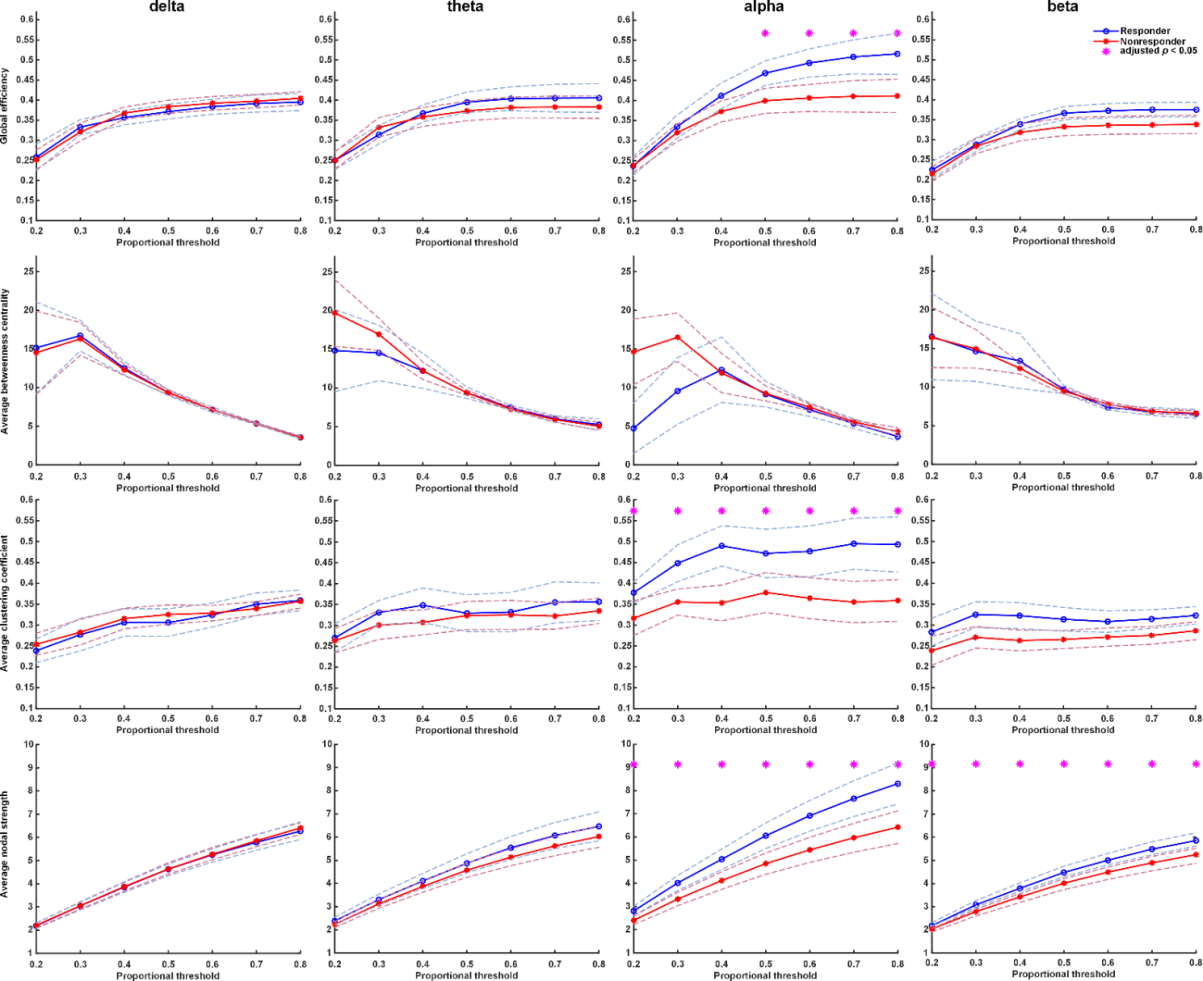
Graph theory-based analyses between responders and non-responders at baseline. The functional connectivity of the non-responsive group was similar to the responsive group at baseline in slow-wave (delta and theta at baseline). In the comparison of functional connectivity in faster waves (alpha and beta bands), it can be observed that the faster wave FC of the treatment-responsive group is higher than that of the non-responsive group at baseline. Asterisks denote statistically significant differences (*: adjusted p under correction for multiple comparisons using the false discovery rate; dashed lines represent the 95% confidence interval)

**Fig. 5:**
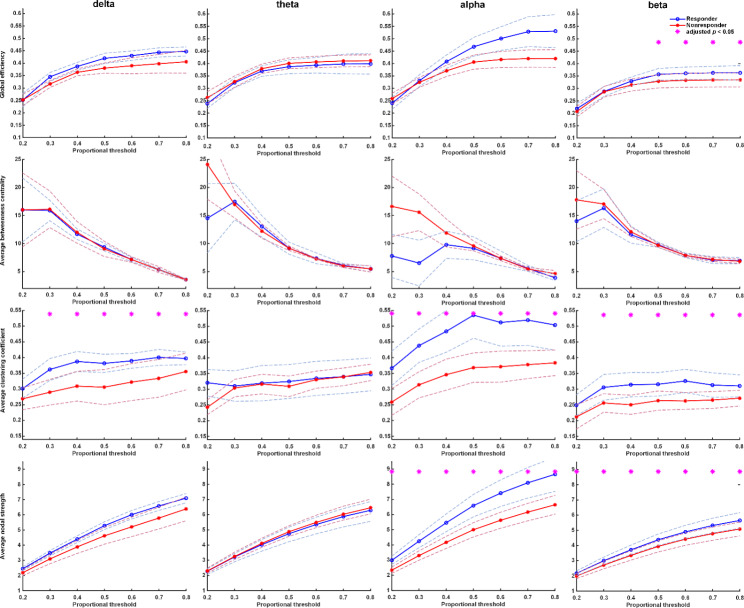
Graph theory-based analyses between responders and non-responders at week-1. The responsive group’s functional connectivity (mean clustering coefficient) significantly increased in the responsive group in the delta wave. In the comparison of functional connectivity in faster waves (alpha and beta bands), it can be observed that the faster wave FC of the treatment-responsive group is still higher than that of the non-responsive group at week-1. Asterisks denote statistically significant differences (*: adjusted p under correction for multiple comparisons using the false discovery rate; dashed lines represent the 95% confidence interval)

**Fig. 6:**
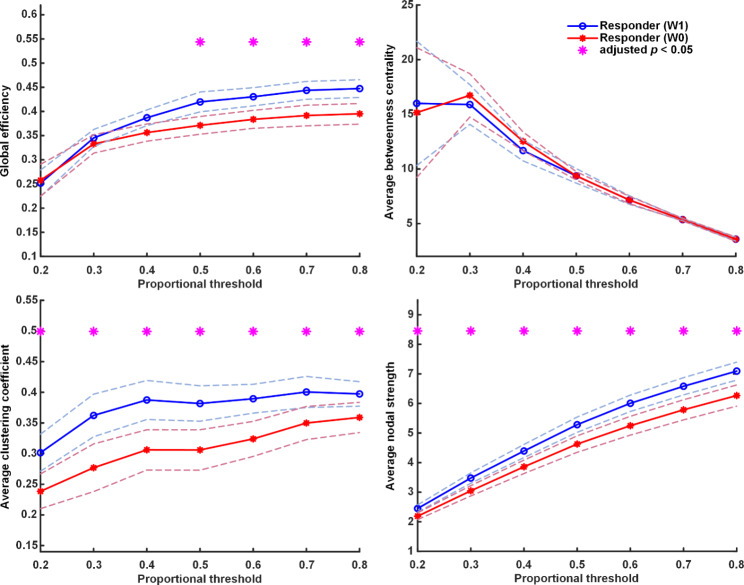
Functional connectivity among responders at baseline and week-1. The results show a significant difference in the global efficiency, mean clustering coefficient, mean node strength and mean betweenness in the delta frequency band. Asterisks denote statistically significant differences (*: adjusted p under correction for multiple comparisons using the false discovery rate; dashed lines represent the 95% confidence interval)

The differences between treatment-responsive and non-responsive groups at baseline are shown in Fig. 4. The FC of the non-responsive group was similar to the responsive group at baseline in slow-wave (delta and theta at baseline). For FC in the faster wave (alpha and beta bands), we observed that the FC of a faster wave of the treatment-responsive group is higher than that of the non-responsive group. Moreover, the results of FC in the responsive and non-responsive groups after one-week treatment indicated that the responsive group also had significantly higher FC in alpha and beta than the non-responsive group (Fig. 5). It was noted that FCs of faster brain waves in the treatment responder group were higher than those in the non-responder group, regardless of whether they were receiving antidepressants treatment. It is worth noting that there was no difference in FC of the delta band between the responders and the non-responders before treatment, while the FC was significantly higher in the responders after treatment. The responsive group’s FC (GE, CC, and NS) showed a significant increase mainly in the delta wave after one week of treatment (Fig. 6). In the non-responsive group, there was no substantial change in the four frequencies band within one week before and after treatment. Early FC change was noted in the responsive group rather than the non-responsive group.

### Correlation between FC and severity of depression

There was no statistical significance for the correlations between the EEG FC values and the HAM-D scores at baseline. Further, for the correlation between EEG After one-week antidepressants treatment, we evaluated correlations between the amount of changes in FC and the amount of change in the HAM-D score at four weeks. The results of mean BC showed a significantly positive correlation while adjusted for age (details in Supplementary Table 2). The more improvement in mean BC during the first week, the more improvement of the patient’s depressive severity over the four weeks of treatment (0.398 in delta band, p < 0.05; 0.420 in theta band, p < 0.05).

### Discriminate ability between SA and FC

In comparing relative power in the four frequency bands between patients and controls at baseline, there was no significant difference, except for the delta wave (0.23 vs. 0.28, p = 0.008) (Supplement Table 3.1). Among patients, there was no significant difference in relative power before and after treatment (Supplementary Table 3.2 and 3.3). Neither did the relative power show differences between responsive and non-responsive groups at baseline and week1, except in higher band frequency (Supplementary Table 3.4 and 3.5). On the other hand, using FC analysis, controls had higher FC values in delta waves than patients before treatment (Fig. 2). After treatment with antidepressants, patients’ FC improved significantly in the delta wave. The FC of treated patients at week1 was close to the FC of controls. In conclusion, FC analysis demonstrated a better discriminative ability for diagnosis and treatment response than SA.

## Discussions

Our results suggest that the greater the FC improvement in the first week, the more reduction of the depressive scores over the four weeks of the treatment period. The FC changes in MDD patients before and after treatment were mainly in the low-frequency (delta) band rather than in the high-frequency band. It’s also noted that the differences in FC between patients before treatment and controls were in the delta band. For the differences between responders and non-responders, we found consistently higher signals in high-frequency waves (alpha and beta bands) in responders than in non-responders over the initial stage of treatment.

Low-frequency band alterations, especially the delta band, have constantly been characterized in depression. One previous meta-analysis of EEG studies showed that MDD patients under an eyes-closed state had significantly increased delta band and theta band activity [33]. Using the network-based-statistic approach, a recent EEG study showed that differences in the delta band exhibit the most discrimination ability for the diagnosis of MDD [34]. Comparing MDD with healthy controls, significantly reduced resting brain connectivity was observed in the delta band in the depressed patient [35]. In addition, synchronization likelihood in the delta frequency bands differentiated depressive patients from controls, with the former exhibiting lower synchronization likelihood than the latter [36]. It was reported that the global phase synchronization index of the depressive patients had a much lower value than controls [37]. Taken together, in the current study, controls and MDD patients exhibited different FC patterns, and altered FC in delta bands characterized MDD patients. Our results in the present study are well supported by previous EEG studies, and FC of the delta band may be a promising marker for assisting the clinical diagnosis of MDD. This study found apparent changes in FC among patients who received medication treatment, which are the combined effects of antidepressants treatment and therapeutic effectiveness. To evaluate the contributions of antidepressants use or therapeutic effects, we investigated FC differences between responders and non-responders. At the initial EEG examination, there were significant differences in FC in high-frequency waves (alpha and beta bands) between the responsive and the non-responsive groups. One early study reported that patients who responded to fluoxetine had more significant EEG alpha signals than non-responders [38], which echoes our findings. Moreover, FC analysis of one week before and after treatment showed that differences in FC persisted in high-frequency bands. These results indicate that higher signals in the alpha and beta band in responders than in non-responders are relatively stable over the initial stage of treatment (i.e., at baseline before treatment and one week after receiving treatment). This is in accordance with the observation of no significant changes in FC before and after treatment among MDD patients in the alpha and beta bands in the present study. An early study also reported no significant EEG changes of alpha power during 12 weeks of treatment with fluoxetine [38]. One early study found that the difference in alpha power activity between treatment responders and non-responders would not change during the treatment course and suggested that alpha power activity represents state-independent characteristics of treatment [38]. High alpha power has been found in recovered depressed patients in a euthymic state, which led Pollock and Schneider [39] to hypothesize that it reflects a specific marker to identify a subgroup of depressed patients with better treatment outcomes. There is an inverse relationship between alpha power and cortical activity [40]. Increased alpha power was evidence of reduced brain activity in depressed patients. Our findings supported this hypothesis, and these features may help differentiate a subgroup of depressed patients who respond to a SSRI. It is known that serotonergic activity is closely related to arousal. In an awake state, serotonergic cells in raphé nuclei display a constant pattern of discharge that decreases in firing rate as arousal decreases to a sleep state [41]. It is possible that increased alpha in depressed patients who respond to an SSRI reflects low arousal associated with low serotonergic activity. Some people may worry about whether the co-existing anxiety will affect the results. We have further put the symptoms of anxiety into the regression model for adjusting, which has not changed the original conclusion of this study. Moreover, the beta power showed a similar difference between responders and non-responders. Previous studies found a positive correlation between beta-band activity and attentional performance [42]. Meanwhile, patients with better cognitive function showed a better response to SSRIs, which provides potential link between beta power and treatment response [43]. Because patients in this study were allowed to take BZD and BZD can influence effects of beta wave, a more complete drug-free study is needed to further explore the role of beta power in predicting treatment response.

A question remains as to why the clinical improvement of depressive severity in SSRI responders did not normalize their alpha power. Although a common serotonergic mechanism might underlie both depression and EEG abnormalities in responders, they may not have the same pharmacological properties. A preclinical study found that the spontaneous firing of serotonin neurons in the dorsal raphé of rats was not altered after two weeks of escitalopram administration [44]. In contrast, combined treatment with escitalopram plus bupropion resulted in a marked increase in firing rates. Moreover, the persistence of alpha abnormalities in treatment responders is compatible with an endophenotypic vulnerability marker to MDD [45]. On the other hand, our findings showed that responders significantly increased FC in the delta band than the non-responders. Further investigation of the physiological roles of the delta band is warranted.

We further investigated the correlation between FC and severity of depression at baseline. A previous EEG study showed a significant negative correlation between FC parameters (degree, efficiency, and betweenness) and HAM-D scores [46]. However, the correlation was not significant in the present study despite the similar magnitude of the correlation coefficient, which is likely due to the moderate sample size to achieve significance. The amount of FC changes is positively correlated with improvement in depression in mean BC, though. The significance level did not pass multiple corrections, which may require further expansion of the sample size to verify the results. Further, this study found that the power SA in the delta band could slightly differentiate the healthy group from the depressive group, but not in other band frequencies. SA cannot distinguish consistently between patients who respond to SSRI treatment and those who do not. The discrimination ability of FC is better than that of the SA in the current study in terms of treatment response. Comparing with FC, which explores the connectivity between nodes, SA targets on the amplitude strength of brain wave. FC is more in line with the functional characteristics of our brain [47]. Because MDD is a mental illness that affects brain function, it is plausible that FC has better discriminative ability than SA.

There are some limitations in the current study. First, the sample size is relatively small in the present study. We may not have sufficient power to detect EEG biomarkers with smaller effect sizes. Second, the benzodiazepine was allowed to use at baseline, which may confound the correlation between EEG marker and treatment response. Third, the background noise was difficult to filter completely by manipulation. While the performance of the denoise and artifact removal function is still limited, we examined the EEG to guarantee that muscle movement, head motion, or channels with poor signal were not involved and selected EEG sections with relatively good quality for further processing and analysis. Fourth, patients in the present study were treated mainly with escitalopram. Therefore, the results may not be inferred for the treatment response in patients treated with other antidepressants. Last, although differences between responders and non-responders in alpha and beta power represent stable, state-independent traits, their biological basis is unknown.

## Conclusions

We found that the FC of the MDD patients at baseline without treatment was worse than that of controls. After one week of treatment, the FC improved and was close to the value of controls. Responders showed better FC in the high-frequency band than non-responders, and this feature exists in both pre-treatment and post-treatment EEG. Signals that change with the treatment process appear primarily in low-frequency FC signals. The improvement of the patient’s FC was positively correlated with the patient’s severity of depression. It is warranted to investigate the clinical usage of FC for depression in the future.

## Electronic supplementary material

Below is the link to the electronic supplementary material.

Supplementary Material 1

## Data Availability

The data will be made available on request. (phkuo@ntu.edu.tw)
