# Supplementary material for: Functional connectivity analysis on electroencephalography signals reveals potential biomarkers for treatment response in major depression
Source: BMC Psychiatry. 2023 Aug 1;23:554. doi: 10.1186/s12888-023-04958-8 (PMC10394892; doi:10.1186/s12888-023-04958-8)
Supplement: Supplementary file 1 — Supplementary Material 1 [file 12888_2023_4958_MOESM1_ESM.docx]

Supplementary Materials

**Exclusion criteria**

Participants were excluded when any of the following criteria were met: 1) current pregnancy or breastfeeding; 2) lifetime history of psychosis; 3) substance dependence or substance abuse; 4) unstable psychiatric or general medical conditions; 5) clinically significant laboratory abnormalities; 6) history of epilepsy or condition requiring an anticonvulsant; 7) any kinds of brain stimulation therapy; 8) medications (psychotropic agents and all medications that could potentially induce seizure); or 9) neurologic disorder (e.g., epilepsy, severe head trauma).

| **Supplementary Table 1. Algorithm formulas of complex network measures.** | | |
| --- | --- | --- |
| Parameters | Annotation | Formula |
| Node Degree | The number of connections that a node has in a network | $k_{i}=\sum_{j\in N} a_{ij}\cdot$ |
| Node strength | The number of connections a node has, and the strength or weight of those connections | $k_{i}^{w}=\sum j\in N^{W}ij\cdot$ |
| Global efficiency | Measures of integration | $E^{W}=\frac{1}{n}\sum i\in N\frac{\sum_{j\in N,j\neq i\left( d_{ij}^{w} \right)} -1}{n-1}\cdot$ |
| Clustering coefficient | Measures of segregation | $C^{W}=\frac{1}{n}\sum i\in N\frac{{2t}_{i}^{w}}{k_{i}\left( k_{i}-1 \right)^{\cdot}}$ |
| Betweenness centrality | Measures of centrality based on node degree or on the length and number of shortest paths between nodes | $b_{i}=\frac{1}{(n-1)(n-2)}\sum_{\begin{aligned} h,j\in N \\ h\neq j,h\neq i,j\neq i \end{aligned}} \frac{\rho_{\mathrm{hj}}(i)}{\rho_{\mathrm{hj}}}$ |
| Basic concepts and notation: N is the set of all nodes in the network, and n is the number of nodes.  (*i*, *j*) is a link between nodes *i* and *j*, (*i*, *j* ∈ N). *a_ij_* is the connection status between *i* and *j*. | | |

| **Supplementary Table 2. The correlation of change in mean betweenness centrality and change in HAM-D after adjusted with age.** | | | | | | | | | | | | | | | | |
| --- | --- | --- | --- | --- | --- | --- | --- | --- | --- | --- | --- | --- | --- | --- | --- | --- |
| Delta band | | | | | Theta band | | | | Alpha band | | | | Beta band | | | |
| PrTh | CC (rho) | CI | | p | CC (rho) | CI | | p | CC (rho) | CI | | p | CC (rho) | CI | | p |
| 0.15 | -0.348 | -0.351 | -0.337 | 0.075 | -0.184 | -0.181 | -0.164 | 0.359 | -0.099 | -0.105 | -0.090 | 0.622 | 0.294 | 0.280 | 0.295 | 0.136 |
| 0.3 | 0.398* | 0.389 | 0.402 | 0.040 | -0.030 | -0.031 | -0.014 | 0.882 | 0.140 | 0.128 | 0.144 | 0.488 | -0.004 | -0.011 | 0.007 | 0.998 |
| 0.45 | 0.108 | 0.101 | 0.116 | 0.592 | 0.279 | 0.269 | 0.285 | 0.158 | 0.226 | 0.215 | 0.233 | 0.256 | 0.029 | 0.016 | 0.032 | 0.884 |
| 0.6 | -0.348 | -0.349 | -0.336 | 0.076 | 0.167 | 0.154 | 0.172 | 0.405 | 0.280 | 0.263 | 0.280 | 0.157 | 0.257 | 0.251 | 0.266 | 0.196 |
| 0.75 | 0.098 | 0.092 | 0.106 | 0.628 | 0.087 | 0.080 | 0.096 | 0.667 | -0.246 | -0.244 | -0.230 | 0.215 | 0.309 | 0.303 | 0.316 | 0.116 |
| 0.9 | 0.156 | 0.146 | 0.161 | 0.436 | 0.420* | 0.413 | 0.426 | 0.029 | 0.131 | 0.121 | 0.134 | 0.516 | 0.262 | 0.250 | 0.266 | 0.187 |
| Asterisks denote statistically significant differences (p < 0.05). Hamilton Rating Scale for Depression (HAM-D); Proportional threshold (PrTh); Correlation coefficient (CC); 95% confidence interval (CI) | | | | | | | | | | | | | | | | |

Supplementary Table 3.1 Comparison of the relative power of EEG for patients at W0, W1, and healthy controls.

|  | Patients at Week-0  (n = 28) | Patients at Week-1  (n = 28) | Healthy Controls  (n = 33) | p*^¥^* | p*^¢^* | p£ |
| --- | --- | --- | --- | --- | --- | --- |
| delta (0.5-4 Hz) | 0.223 ± 0.064 | 0.233 ± 0.064 | 0.275 ± 0.089 | 0.616 | 0.008* | 0.045* |
| theta (4-8 Hz) | 0.158 ± 0.058 | 0.154 ± 0.057 | 0.146 ± 0.028 | 0.509 | 0.800 | 0.925 |
| alpha (8-13 Hz) | 0.267 ± 0.083 | 0.253 ± 0.073 | 0.255 ± 0.070 | 0.072 | 0.680 | 0.960 |
| beta (13-30 Hz) | 0.365 ± 0.094 | 0.372 ± 0.098 | 0.335 ± 0.065 | 0.585 | 0.167 | 0.063 |

*p^¥^*: W1 vs. W0 (Wilcoxon signed-rank test);*p^¢^*: W0 vs. Control (Wilcoxon rank-sum test);

*p^£^*: W1 vs. Control (Wilcoxon rank-sum test).

Supplementary Table 3.2 Comparison of the relative power of EEG for responders at W0 and W1.

|  | W0_R (n = 13) | W1_R (n = 13) | p*^¥^* |
| --- | --- | --- | --- |
| delta (0.5-4 Hz) | 0.229 ± 0.078 | 0.245 ± 0.071 | 0.735 |
| theta (4-8 Hz) | 0.154 ± 0.035 | 0.156 ± 0.037 | 0.588 |
| alpha (8-13 Hz) | 0.298 ± 0.083 | 0.285 ± 0.070 | 0.244 |
| beta (13-30 Hz) | 0.332 ± 0.053 | 0.326 ± 0.059 | 0.946 |

*p^¥^*: W1_R vs. W0_R (Wilcoxon signed-rank test);

Supplementary Table 3.3 Comparison of the relative power of EEG for non-responders at W0 and W1.

|  | W0_NR (n = 15) | W1_NR (n = 15) | p*^¥^* |
| --- | --- | --- | --- |
| delta (0.5-4 Hz) | 0.219 ±0.051 | 0.222 ±0.058 | 0.804 |
| theta (4-8 Hz) | 0.161 ±0.074 | 0.153 ±0.071 | 0.169 |
| alpha (8-13 Hz) | 0.239 ±0.075 | 0.225 ±0.065 | 0.252 |
| beta (13-30 Hz) | 0.393±0.113 | 0.412 ±0.110 | 0.389 |

*p^¥^*: W1_NR vs. W0_NR (Wilcoxon ssigned-ranktest);

Supplementary Table 3.4 Comparison of the relative power of EEG for responders and non-responders at W0.

|  | W0_R (n = 13) | W0_NR (n = 15) | p *^¶^* |
| --- | --- | --- | --- |
| delta (0.5-4 Hz) | 0.229 ± 0.078 | 0.219 ± 0.051 | 0.890 |
| theta (4-8 Hz) | 0.154 ± 0.035 | 0.161 ± 0.074 | 0.747 |
| alpha (8-13 Hz) | 0.298 ± 0.083 | 0.239 ± 0.075 | 0.088 |
| beta (13-30 Hz) | 0.332 ± 0.053 | 0.393± 0.113 | 0.038* |

*p^¶^*: W0_R vs. W0_NR (Wilcoxon rank-sum test);

Supplementary Table 3.5 Comparison of the relative power of EEG for responders and non-responders at W1.

|  | W1_R (n = 13) | W1_NR (n = 15) | p *^ñ^* |
| --- | --- | --- | --- |
| delta (0.5-4 Hz) | 0.245 ± 0.071 | 0.222 ± 0.058 | 0.490 |
| theta (4-8 Hz) | 0.156 ± 0.037 | 0.153 ± 0.071 | 0.333 |
| alpha (8-13 Hz) | 0.285 ± 0.070 | 0.225 ± 0.065 | 0.027* |
| beta (13-30 Hz) | 0.326 ± 0.059 | 0.412 ± 0.110 | 0.006* |

*p^ñ^*: W1_R vs. W1_NR (Wilcoxon rank-sum test).

**Exclusion criteria**

1) current pregnancy; 2) lifetime history of psychosis; 3) substance dependence in the past six months or substance abuse in the past two months; 4) unstable psychiatric or general medical conditions; 5) clinically significant laboratory abnormalities; 6) history of epilepsy or condition requiring an anticonvulsant; 7) ECT; 8) VNS, or TMS in the current episode; 9) medications (psychotropic agents and all medications that could potentially induce seizure); 10) current psychotherapy; 11) significant suicide risk; or (12) neurologic disorder

**Outpatients with MDD and HCs**

(n=28 vs 33)

**Study steps**

1. Washout period for antidepressants: 7-10days
2. Baseline exam:

HAMD, blood exam, EEG

1. Regular follow-up:

w1 EEG; w1 w2 w4 w6 w8 m6 m12 HAM-D

Aim 1: To study the differences of FC between HCs and patients and the differences between MDD patients before treatment and after treatment (under the effect of antidepressants and therapeutic effects).

Aim 2: To investigate the true correlation between FC and treatment response (excluding the effects of antidepressants), we compare the change of FC among treatment responsive and non-responsive group

Aim 3: To investigate the correlation between the severity of MDD and FC in EEG data.

Aim 4: To investigate the discriminative ability of diagnosis and treatment response between band power value and FC.

Non-responsive group in w4

**Treatment response:**

HAM-D score (≥ 50% improvement)

Responsive group in w4

Previous study: outpatients with depression and control (N=402)

8 weeks f/u

Responsive group in w8

Non-responsive group in w8

Potential gene and gene set of treatment response (Aim3,5)

Polygenic score of treatment response Network analyses (Aim4) GWAS study (aim3)

PRS (aim4)

2^nd^ part: a genetic study

**Supplementary Figure 1. Study flowchart and study aim**

Abbreviations: TRD, treatment-resistant depression; MDD, major depressive disorder; HCs, healthy controls; ECT, electroconvulsive therapy; VNS, vagal nerve stimulation; TMS, transcranial magnetic stimulation; HAM-D, Hamilton Rating Scale for Depression; SA, spectrum analysis; FC, functional connectivity

**
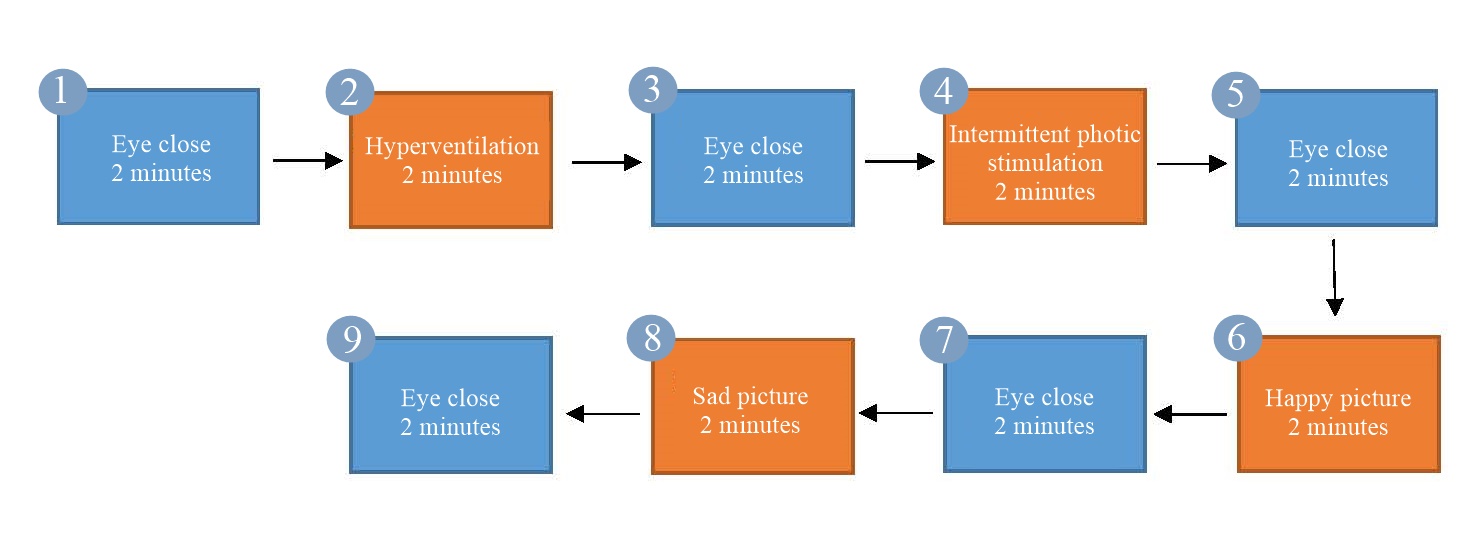
Supplementary Figure 2. Step of EEG examination**
